# Cutaneous and mucosal melanoma among the Palestinian population: a retrospective clinicopathological study with comparative regional context

**DOI:** 10.1186/s12885-026-16393-5

**Published:** 2026-06-29

**Authors:** Ibrahim Salhi, Sami Bannoura, Orgina Abedaljawwad, Zaidoun Salah

**Affiliations:** Medicare Diagnostic Laboratories, West Bank, Palestine

**Keywords:** Melanoma, Cutaneous melanoma, Mucosal melanoma, Acral melanoma, Palestine, Eastern Mediterranean, Clinicopathology, Histopathology, Cancer registry, AJCC staging

## Abstract

**Background:**

Cutaneous and mucosal melanoma are biologically and clinically distinct malignancies whose epidemiology varies markedly across populations and skin phototypes. In Arab and other Eastern Mediterranean populations, melanoma is comparatively uncommon but is frequently diagnosed at an advanced stage and shows a relatively high proportion of acral and mucosal subtypes. Data describing melanoma in the Palestinian population are scarce. We characterised the clinicopathological profile of cutaneous and mucosal melanoma diagnosed at a large Palestinian diagnostic pathology service and placed the findings in a comparative regional context.

**Methods:**

We performed a retrospective review of all histopathologically confirmed cases of cutaneous and mucosal melanoma reported at Medicare Diagnostic Laboratories, West Bank, Palestine, between May 2008 and May 2026. Demographic variables (age, sex), anatomical site, melanoma subtype, depth of invasion (Clark level), and, where recorded, Breslow thickness and American Joint Committee on Cancer (AJCC) stage were abstracted from pathology records. Diagnoses were confirmed on haematoxylin-eosin sections supported by immunohistochemistry (S100, SOX10, HMB-45, Melan-A). Repeat specimens were consolidated to the patient level, and findings were compared with published series from neighbouring countries.

**Results:**

Fifty-seven patients with primary melanoma were identified, comprising 47 cutaneous (82.5%) and 10 mucosal (17.5%) tumours. The mean age at diagnosis was 59.5 +/- 17.0 years (range 10–87), and there was a slight female predominance (30 women, 52.6%; male-to-female ratio 0.90:1). Acral sites (sole, heel, plantar, and subungual regions) accounted for 15 of 47 cutaneous melanomas (31.9%), the most common single location, followed by the head and neck (14, 29.8%). Among mucosal tumours, the anorectum was the predominant site (6 of 10, 60%). A histological subtype was explicitly recorded in only 19 of 57 cases; within this limited subset the nodular pattern predominated (18 of 19), although the large proportion of cases without a stated subtype precludes firm conclusions about the true distribution of melanoma subtypes in this cohort. The Clark level was documented in 27 patients, of whom 26 (96.3%) had level IV-V (deep) invasion. A further 17 patients presented with metastatic melanoma deposits without a primary tumour in the archive.

Melanoma in this Palestinian cohort was characterised by a slight female predominance, a high proportion of acral (31.9% of cutaneous) and mucosal (17.5% of all primary) tumours, a predominantly nodular phenotype among the subset of cases with a recorded subtype, and deep invasion at diagnosis. This profile mirrors patterns reported in neighbouring Arab and Eastern Mediterranean populations and contrasts with more lightly pigmented (Fitzpatrick I–II) Western populations. The findings underline the need for improved melanoma awareness, routine examination of acral and mucosal sites, and strengthened cancer registration in Palestine.

## Background

Melanoma is an aggressive malignancy arising from melanocytes and is responsible for the majority of skin-cancer deaths despite accounting for only a minority of skin-cancer diagnoses. According to GLOBOCAN 2020, an estimated 325,000 new cases of cutaneous melanoma and 57,000 related deaths occurred worldwide, and the global burden is projected to rise substantially over the coming decades [1, 2]. Incidence is highest in lightly pigmented populations (Fitzpatrick skin phototypes I–II) of Australia, North America, and Northern Europe, where the risk of cutaneous melanoma on sun-exposed skin rises with cumulative and intermittent ultraviolet exposure [3, 4]. This ultraviolet-driven relationship applies principally to melanomas arising on sun-exposed skin; acral and mucosal melanomas, by contrast, are largely independent of ultraviolet exposure and do not share this aetiology, as discussed below [5, 6]. The clinical and biological behaviour of melanoma, the distribution of histological subtypes, and the stage at which patients present differ considerably between populations and skin phototypes [5].

In more darkly pigmented individuals (Fitzpatrick skin phototypes IV–VI) and in non-European populations, melanoma is far less common but disproportionately affects sun-protected sites. Acral lentiginous melanoma, arising on the palms, soles, and subungual regions, represents only 1–3% of melanomas in lightly pigmented (Fitzpatrick I–II) populations but constitutes a much larger share among people of African, Asian, Hispanic, and Middle Eastern ancestry, and is frequently associated with delayed diagnosis and poorer outcomes [5, 7]. The 2018 World Health Organization classification formally recognises melanoma as a family of distinct entities defined by their degree of cumulative sun damage and evolutionary pathway, encompassing low-ultraviolet pathways such as acral and mucosal melanoma [6].

Mucosal melanoma is a rare and especially aggressive entity, accounting for less than 1% of all melanomas and arising from melanocytes of the mucosal linings of the head and neck, anorectal region, and female genital tract [8, 9]. It is characterised by occult anatomical locations, late presentation, high rates of regional and distant spread, and a dismal prognosis, with reported five-year survival of only 10–25% [9–11]. Because mucosal and acral melanomas are comparatively over-represented in populations where ultraviolet-driven cutaneous melanoma is rare, the melanoma profile of Eastern Mediterranean and Arab populations is expected to differ from that of Western series [5, 7, 12].

In Palestine, cancer is the second leading cause of death after cardiovascular disease, and the cancer burden is rising against a backdrop of a constrained and fragmented health system [13, 14]. A national cancer registry was established in 1998 with separate operations in the West Bank and the Gaza Strip; nonetheless, under-reporting, fragmented referral pathways, and limited access to specialised oncology care continue to compromise the completeness of cancer surveillance, and many Palestinian patients are referred abroad for diagnosis and treatment [13, 15, 16]. Within this context, melanoma represents a small fraction of recorded cancers, and its clinicopathological characteristics in the Palestinian population have not been systematically described.

Neighbouring countries provide an informative comparative frame. Series from Turkey, Lebanon, Saudi Arabia, and from Jordanian referral centres treating Palestinian patients consistently report younger age at diagnosis, frequent thick tumours, a notable proportion of acral disease, and advanced stage at presentation relative to Western cohorts [12, 16–18]. Characterising melanoma in the Palestinian population is therefore important both for local cancer control and for understanding melanoma biology across the Eastern Mediterranean. We undertook a retrospective clinicopathological study of cutaneous and mucosal melanoma diagnosed at a large Palestinian diagnostic pathology service and interpreted the findings alongside published regional data.

## Methods

### Study design and setting

This was a retrospective, laboratory-based observational study conducted at Medicare Diagnostic Laboratories, a high-volume private histopathology and diagnostic service in the West Bank, Palestine, that receives specimens from multiple hospitals, clinics, and dermatology and surgical practices across the territory. The laboratory information system was searched to identify all cases reported with a histopathological diagnosis of melanoma between May 2008 and May 2026.

### Case selection

All histopathologically confirmed primary cutaneous and mucosal melanomas reported during the study period were eligible for inclusion. Cases were identified through a systematic search of the laboratory information system using diagnostic codes and free-text terms for melanoma. Benign and non-melanoma diagnoses retrieved by the text search, such as melanonychia, melanocytic naevi, and non-melanoma skin cancers, and specimens reported as negative for melanoma were excluded. Multiple specimens belonging to the same individual (for example, an initial biopsy with a subsequent re-excision or a nodal metastasis) were consolidated so that each patient was counted once. Primary cutaneous and primary mucosal melanomas formed the main analytic cohort, while specimens showing only metastatic deposits without a primary tumour in the archive were tabulated separately. Where diagnostic ambiguity existed, the original slides were re-reviewed by a consultant pathologist.

### Histopathological assessment

Diagnoses were established on formalin-fixed, paraffin-embedded tissue stained with haematoxylin and eosin and were supported, where required, by an immunohistochemical panel including S100, SOX10, HMB-45, and Melan-A. For each case, the following variables were recorded when available: patient age and sex; anatomical site; clinical specimen type; melanoma subtype (superficial spreading, nodular, lentigo maligna, acral lentiginous, spindle cell, and mucosal); depth of invasion (Clark level); Breslow thickness; presence of ulceration; and resection-margin status. Tumours were classified according to the 2018 World Health Organization classification of melanocytic tumours [6] and, where data permitted, staged using the eighth edition of the American Joint Committee on Cancer (AJCC) melanoma staging system [19]. Because Breslow thickness and AJCC stage were not uniformly documented across the full archival period, the Clark level of invasion was used as the consistently available measure of tumour depth. We acknowledge that the Clark level is not a component of the eighth-edition AJCC staging system and has inferior prognostic value to Breslow thickness, which is the preferred measure of tumour depth; it is reported here only because it was the depth parameter most consistently available in the historical archive, and the resulting incompleteness of Breslow thickness, subtype, and stage data is acknowledged as a limitation.

### Data management and statistical analysis

De-identified data were extracted into a structured spreadsheet and verified against the original pathology reports. Continuous variables are summarised as mean +/- standard deviation or median with range, and categorical variables as frequencies and percentages. Subgroups (cutaneous versus mucosal; male versus female) were compared descriptively. Findings were then compared with published melanoma series from neighbouring countries to provide regional context.

### Ethics

The study was approved by the Institutional Review Board of Medicare Diagnostic Laboratories (approval number 2026-HIS-321-7) and was conducted in accordance with the principles of the Declaration of Helsinki. Because the analysis used de-identified archival pathology data, the Board waived the requirement for individual informed consent.

## Results

### Cohort and demographic characteristics

**Fig. 1:**
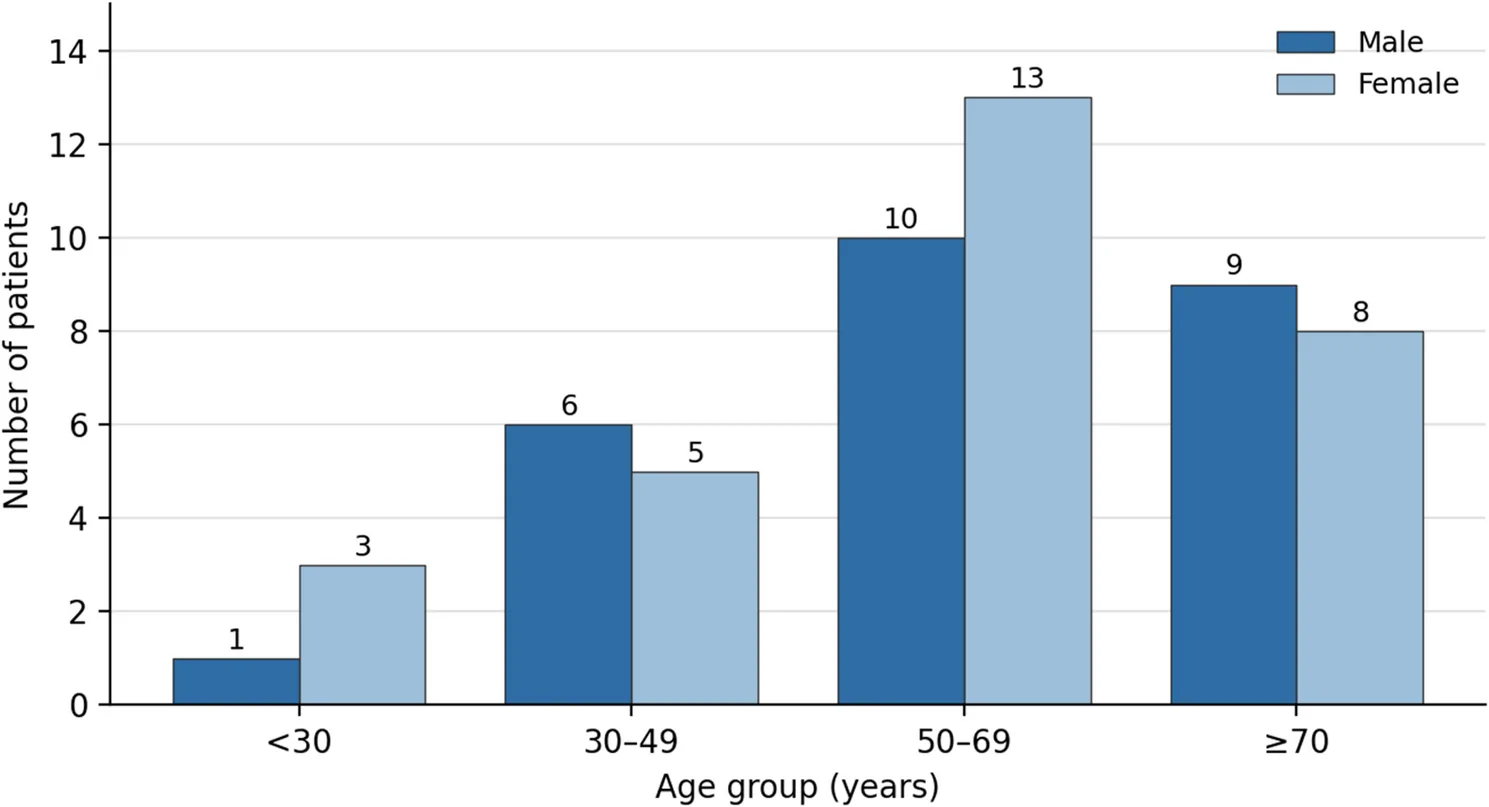
Distribution of melanoma patients by age group and sex (*n* = 57). Number of primary melanoma patients in each age group, stratified by sex; most patients were diagnosed in the fifth to seventh decades

After consolidation of repeat specimens, 57 patients with primary melanoma were identified over the study period. Cutaneous melanoma accounted for 47 cases (82.5%) and mucosal melanoma for 10 cases (17.5%). The mean age at diagnosis was 59.5 +/- 17.0 years (median 61, range 10–87); patients with mucosal disease were on average older (mean 63.6 years) than those with cutaneous disease (mean 58.6 years). Thirty patients (52.6%) were women and 27 (47.4%) were men, giving a male-to-female ratio of 0.90:1. Most patients were diagnosed in the fifth to seventh decades of life. In addition to the 57 primary cases, a further 17 patients presented with metastatic melanoma deposits, most often in regional lymph nodes or viscera, without a primary tumour represented in the archive; detailed and systematic data on the metastatic site, age, and sex of these archival metastatic-only specimens were not uniformly retrievable, and they are tabulated separately and excluded from the primary analytic cohort. The baseline characteristics are summarised in Table 1, and the distribution of cases by age group and sex is shown in Fig. 1.

**Table 1:** Demographic characteristics of the primary melanoma cohort (*n* = 57)

Characteristic	Category	*n* (%)
Total primary cases	—	57 (100)
Sex	Male	27 (47.4)
	Female	30 (52.6)
Age group (years)	< 30	4 (7.0)
	30–49	11 (19.3)
	50–69	23 (40.4)
	≥ 70	17 (29.8)
	Not recorded	2 (3.5)
Mean age ± SD (years)	Median 61; range 10–87	59.5 ± 17.0
Melanoma category	Cutaneous	47 (82.5)
	Mucosal	10 (17.5)

Male-to-female ratio 0.90:1. An additional 17 patients presented with metastatic deposits without a primary tumour in the archive and are not included in the primary cohort

*SD* Standard deviation

### Anatomical distribution and histological subtype

**Fig. 2:**
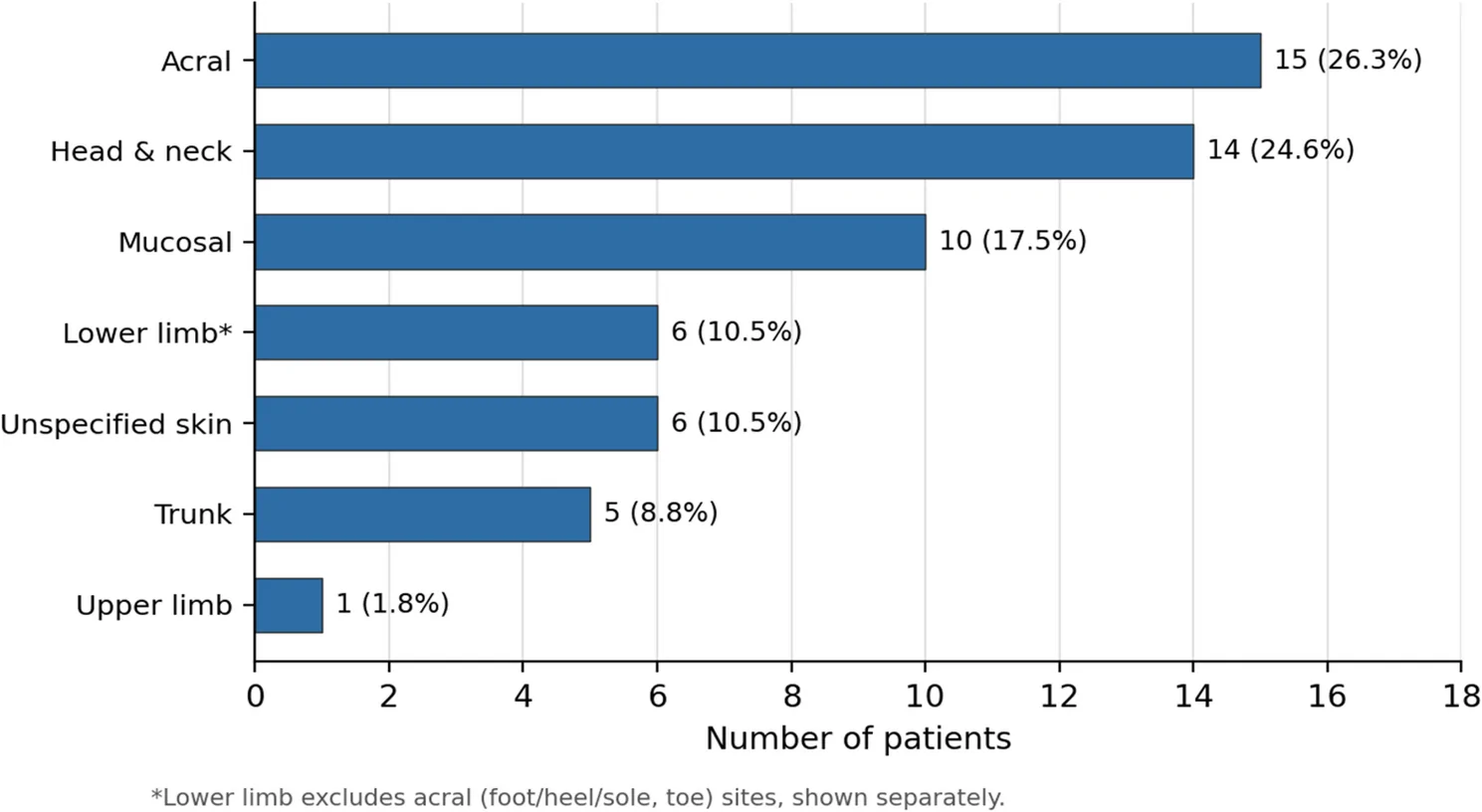
Anatomical distribution of primary melanoma (*n* = 57). Frequency of primary melanoma by anatomical site. Acral sites (sole, heel, plantar surface, subungual) were the most common location overall; mucosal tumours are grouped together; lower limb excludes acral sites

Acral sites, the sole, heel, plantar surface, and subungual regions, were the single most common location, accounting for 15 of 47 cutaneous melanomas (31.9%), followed by the head and neck (14, 29.8%), the non-acral lower limb (6, 12.8%), the trunk (5, 10.6%), and the upper limb (1, 2.1%); the site was not specified for 6 cutaneous cases (12.8%). Among the 10 mucosal melanomas, the anorectum was the predominant primary site (6, 60%), followed by the vulvovaginal tract (2, 20%), the sinonasal cavity (1, 10%), and the oral cavity/oropharynx (1, 10%). The overall anatomical distribution is presented in Fig. 2. A histological subtype was explicitly recorded in 19 cases; of these, the nodular pattern overwhelmingly predominated (18 of 19, 94.7%, including one amelanotic nodular tumour), with a single spindle-cell melanoma. No case was explicitly classified as superficial spreading or lentigo maligna melanoma; however, a subtype was not stated for the remaining 38 of 57 cases (66.7%), and the descriptor “nodular” in many reports denotes the predominant pattern of vertical growth rather than a formal subtype assigned after assessment of the in situ (radial) component. Because the large majority of cases lacked a recorded subtype, and because acral tumours were classified by anatomical site rather than by subtype (so that a proportion are likely to represent acral lentiginous melanoma), no firm conclusion can be drawn about the true prevalence of individual melanoma subtypes in this cohort; it is also possible that, by local reporting convention, a subtype was recorded only when it differed from conventional superficial spreading melanoma, which would further bias the apparent distribution. The distribution of subtypes and core tumour characteristics is detailed in Table 2.

**Table 2:** Anatomical, histological, and depth characteristics of primary melanoma (*n* = 57)

Variable	Category	*n* (%)
Anatomical site	Acral (sole/heel/plantar, subungual)	15 (26.3)
	Head & neck	14 (24.6)
	Mucosal	10 (17.5)
	Lower limb (non-acral)	6 (10.5)
	Unspecified skin	6 (10.5)
	Trunk	5 (8.8)
	Upper limb	1 (1.8)
Mucosal subsite (*n* = 10)	Anorectal	6 (60.0)
	Vulvovaginal	2 (20.0)
	Sinonasal	1 (10.0)
	Oral / oropharyngeal	1 (10.0)
Histological subtype	Nodular (incl. 1 amelanotic)	18 (31.6)
	Spindle cell	1 (1.8)
	Superficial spreading	0 (0)
	Lentigo maligna	0 (0)
	Not otherwise specified	38 (66.7)
Clark level (*n* = 27 graded)	III	1 (3.7)
	IV	22 (81.5)
	V	4 (14.8)

Percentages for anatomical site, subtype, and category are of all 57 primary cases; mucosal subsite is of 10 mucosal cases; Clark level is of the 27 patients in whom level was recorded. Acral tumours represented 31.9% of the 47 cutaneous melanomas. Breslow thickness and AJCC stage were not uniformly documented across the archival period; Clark level was used as the consistently available measure of invasion depth. Because a histological subtype was recorded in only 19 of 57 cases, the subtype frequencies shown should be interpreted with caution and do not establish the true subtype distribution in the cohort

### Depth of invasion at diagnosis

**Fig. 3:**
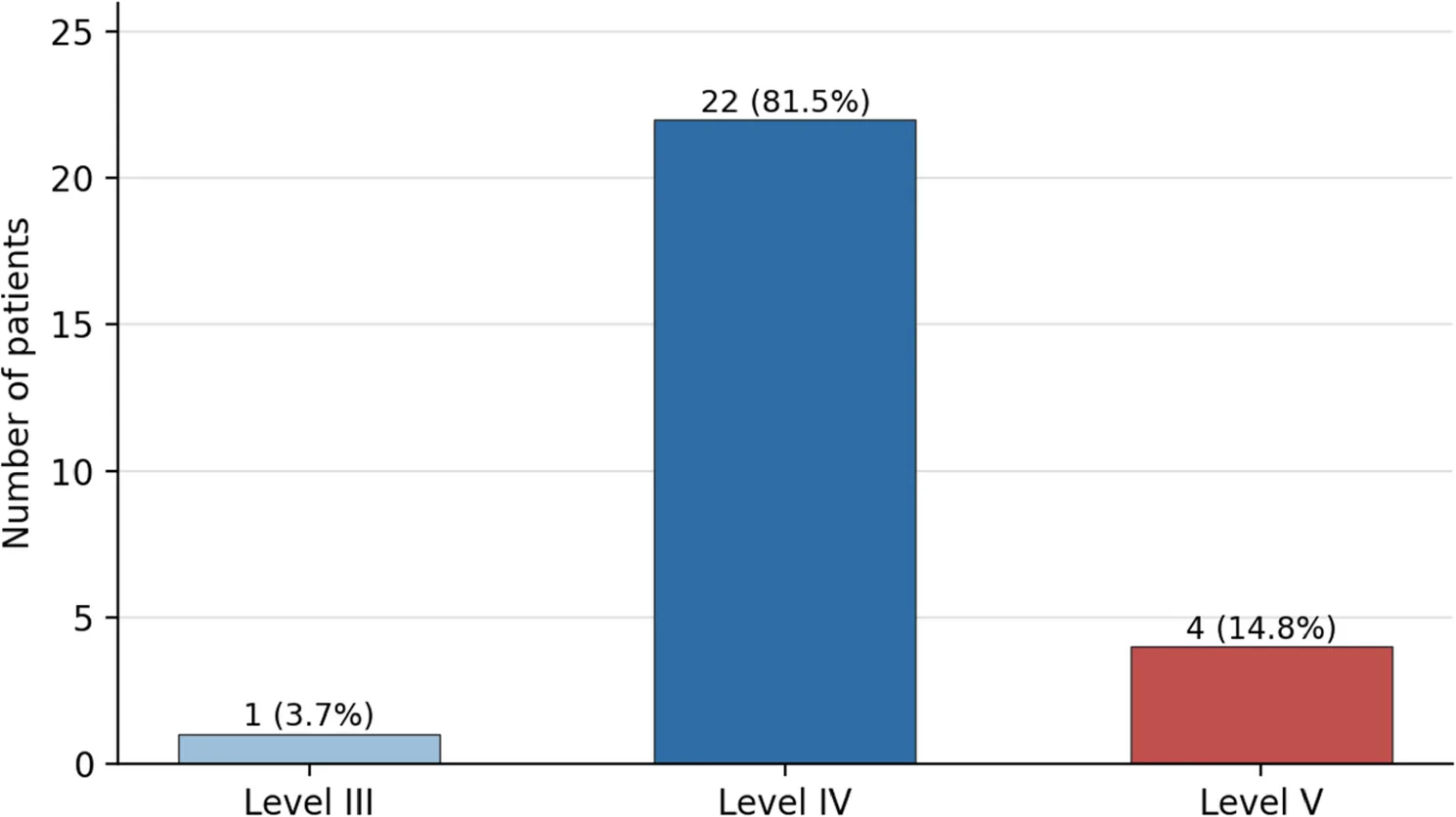
Depth of invasion (Clark level) at diagnosis (*n* = 27 with level recorded). Clark level of invasion among the 27 patients in whom level was documented; 96.3% were Clark level IV-V, indicating deep invasion at diagnosis

**Fig. 4:**
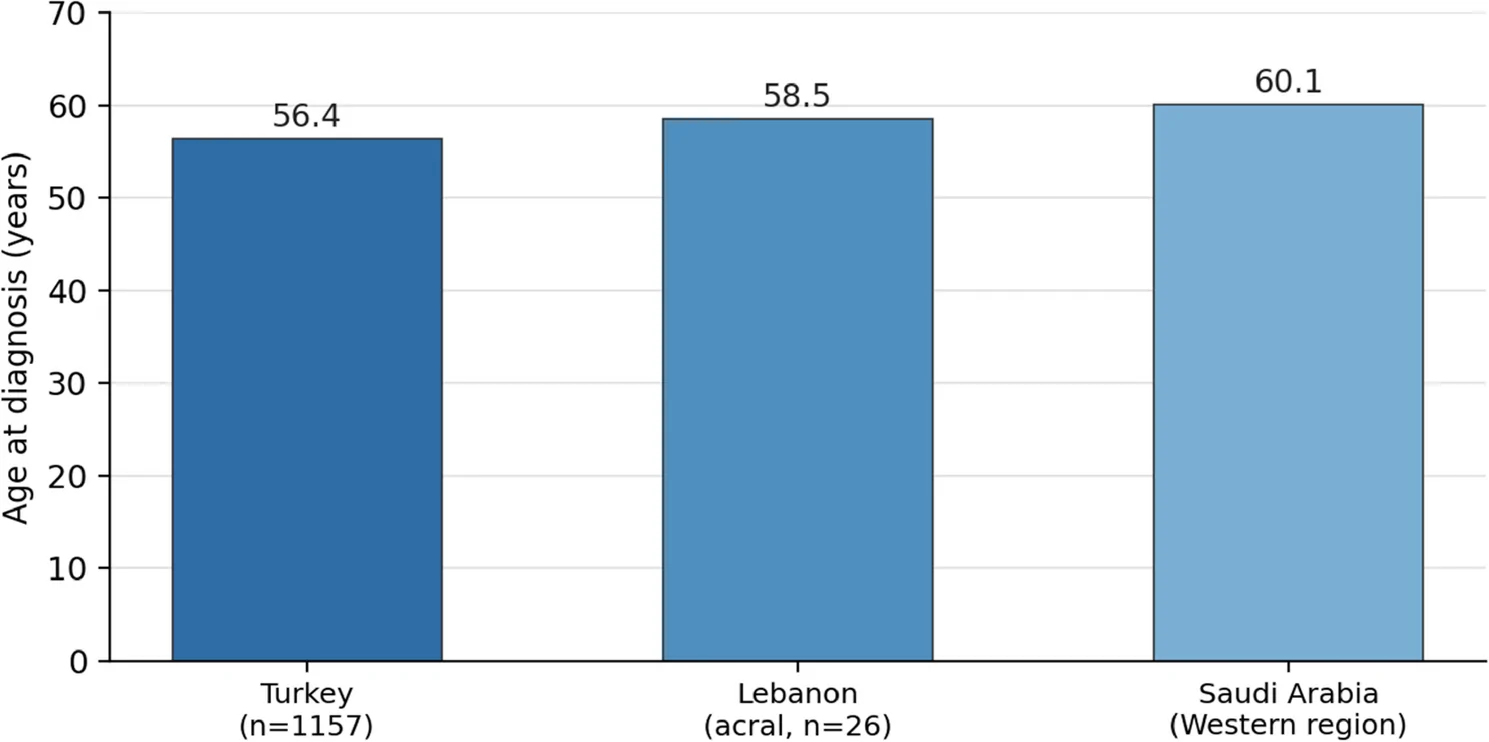
Reported age at melanoma diagnosis: present Palestinian cohort versus neighbouring series. Reported age at melanoma diagnosis in the present cohort (mean 59.5 years) compared with published series from Turkey (median 56.4 y [17]), Lebanon (acral, median 58.5 y [12]), and Saudi Arabia (mean approximately 60.1 y [18]). External values are drawn from the cited studies and shown for comparative context.

The Clark level of invasion was documented in 27 patients. Invasion was deep in the great majority: 22 patients (81.5%) had Clark level IV and 4 (14.8%) had Clark level V, so that 26 of 27 graded tumours (96.3%) were Clark level IV-V, with only a single level III tumour (Fig. 3). Breslow thickness was recorded numerically in a minority of reports (for example, 4 mm and 18 mm in two nodular tumours), consistent with deep invasion. Breslow thickness, the single most important histological prognostic factor in melanoma, was available as a recorded numerical value in only a small minority of reports; this incompleteness reflects the absence of synoptic (structured) reporting during much of the archival period and is acknowledged as a limitation, and the routine adoption of synoptic melanoma reporting is recommended so that Breslow thickness is captured for every future case. This advanced depth at presentation, together with the high proportion of acral and mucosal disease, is summarised against published regional series in Table 3, and the reported age at diagnosis across selected neighbouring Eastern Mediterranean series is illustrated for comparison in Fig. 4.

**Table 3:** Comparative clinicopathological data: present Palestinian cohort and neighbouring countries (published series)

Population / setting	Study (reference)	Period / size	Key findings
Palestine — present study	Salhi et al. (this study)	2008–2026; *n* = 57	47 cutaneous (82.5%), 10 mucosal (17.5%); slight female predominance (M: F 0.90:1); mean age 59.5 y; acral 31.9% of cutaneous; nodular phenotype predominant with no SSM/LMM recorded; Clark IV–V in 96.3% of graded tumours.
Palestinian patients, Jordan (KHCC)	Mansour et al. 2022 [13]	Referral cohort	Cross-border referral of Palestinian cancer patients to Jordan; care frequently delivered outside the territory, complicating local registration and follow-up.
Turkey	Abali et al. 2015 [15]	*n* = 1157	Median age 56.4 y; 55.8% male; lower-extremity most common site (30.5%); superficial spreading 30.9%; frequent thick (> 4 mm) tumours; stage III 56.6%, stage IV 19.6%.
Lebanon (acral melanoma)	Charbel et al. 2025 [16]	26 of 331 melanomas	Acral = 8% of all melanomas (vs. 1–3% in Western series); median age 58.5 y; 54% female; 96% Middle-Eastern origin; 81% plantar.
Saudi Arabia (Western region)	Albasri & Borhan 2018 [17]	11-year series	Melanoma ≈ 3.7% of skin cancers; male-to-female ratio 2.2:1; mean age ≈ 60 y.
Mucosal melanoma (reference cohorts)	Clavero-Rovira 2024 [18]; Carbo-Bague 2022 [19]	Cohort / population-based	Mucosal melanoma < 1% of all melanomas; head-and-neck, anorectal, and genital sites; nodal or distant spread in ≈ 30% at diagnosis; markedly poorer survival than cutaneous melanoma.

Reference numbers correspond to the reference list

*KHCC* King Hussein Cancer Center, *SSM* Superficial spreading melanoma, *LMM* Lentigo maligna melanoma

## Discussion

To our knowledge, this is among the first dedicated clinicopathological descriptions of cutaneous and mucosal melanoma in the Palestinian population. Across 57 primary cases, the overall picture, a slight female predominance, acral involvement in nearly a third of cutaneous tumours, mucosal melanoma comprising 17.5% of primary disease, a near-exclusive nodular phenotype, and Clark level IV-V invasion in 96% of graded tumours, is consistent with the broader pattern reported across Arab and Eastern Mediterranean populations and stands in clear contrast to fair-skinned Western series, in which superficial spreading melanoma on intermittently sun-exposed skin predominates [3, 5, 7].

The low relative frequency of melanoma in the Palestinian population is consistent with regional epidemiology. Melanoma represents well under 1% of recorded cancers in the Palestinian population [13], and it forms only a small share of skin malignancies in neighbouring Arab settings; in a western Saudi series, for example, melanoma accounted for fewer than 4% of skin cancers [18]. This pattern reflects the strong influence of skin phototype on ultraviolet-driven melanoma risk: constitutional pigmentation, rather than latitude or ambient ultraviolet exposure alone, is the dominant determinant of cutaneous melanoma incidence, so that more darkly pigmented (Fitzpatrick IV–VI) Eastern Mediterranean populations experience substantially lower rates than more lightly pigmented (Fitzpatrick I–II) Western populations [5, 7].

Despite its lower incidence, melanoma in our cohort and across the region is marked by unfavourable presentation. Series from neighbouring countries consistently report younger age at diagnosis, frequent thick tumours, and advanced stage relative to Western data. The Melanoma Turkish Study of 1,157 patients documented a median age of 56 years, lower-extremity predominance, frequent thick (> 4 mm) tumours, and a notable burden of acral lentiginous disease, features attributed to low melanoma awareness and delayed presentation [17]. Turkish, Saudi, and Lebanese cohorts similarly describe a male predominance and a mean age around the sixth decade [12, 17, 18]. Our mean age of 59.5 years sits squarely within this range, and the deep Clark level IV-V invasion seen in 96% of graded tumours reinforces the interpretation that, where melanoma is perceived as rare, both public and professional vigilance are reduced, allowing tumours to reach a greater depth before diagnosis [15, 17].

A defining feature of melanoma in pigmented populations is the relative over-representation of acral disease, and this is increasingly documented across the Middle East and North Africa. A 12-year Lebanese series found that acral melanoma comprised 8% of all melanomas, substantially higher than the 1–3% reported in European and North American cohorts, with most tumours arising on the plantar surface and a median age in the late fifties [12]. The acral burden in our cohort was higher still: acral sites accounted for 31.9% of cutaneous melanomas and were the single most common location overall, the majority arising on the sole, heel, or plantar surface. Because acral sites are infrequently examined and lesions may be mistaken for benign conditions or trauma, acral melanoma is prone to diagnostic delay in exactly the populations where it is proportionally most common [5, 7, 12]. Systematic inspection of the palms, soles, and nail units should therefore be emphasised in clinical practice and in melanoma-awareness messaging in Palestine. As noted above, these acral tumours were classified primarily by anatomical site, and a specific subtype (such as acral lentiginous melanoma) was not separately recorded in most cases.

Mucosal melanoma, although rare everywhere, carries a disproportionate clinical impact and merits specific attention in our setting. Population-based and cohort studies confirm that mucosal melanoma accounts for under 1% of melanomas, arises most often in the head and neck, anorectal, and genital regions, presents with nodal or distant spread in roughly a third of patients, and is associated with markedly poorer survival than cutaneous melanoma [8–11]. In our series, mucosal melanoma made up 17.5% of all primary melanomas, far above the under-1% share seen in Western incidence data, with the anorectum the leading primary site (60% of mucosal cases) followed by the vulvovaginal, sinonasal, and oral/oropharyngeal regions. This elevated proportion is best understood not as a high absolute incidence but as the arithmetic consequence of a small denominator of ultraviolet-driven cutaneous melanoma combined with referral of symptomatic mucosal tumours, and it reinforces the need to keep mucosal melanoma in the differential for pigmented or non-pigmented mucosal lesions [6, 9, 11]. It is also notable that mucosal melanoma constitutes a higher proportion of all melanomas in Asian populations than in Western populations, a difference attributed largely to the lower background incidence of ultraviolet-driven cutaneous melanoma in more darkly pigmented groups; the elevated mucosal share observed in our cohort is consistent with this broader pattern [5, 9].

The findings carry practical implications for cancer control in Palestine. The advanced depth at presentation observed here and regionally is amenable to intervention through earlier detection, and similar Eastern Mediterranean settings have called for strengthened public awareness, training of primary-care and dermatology providers in the recognition of acral and mucosal lesions, and investment in complete, high-quality cancer registration [13, 15, 16]. Because a substantial share of Palestinian cancer patients are diagnosed or treated through referral to centres such as the King Hussein Cancer Center in Jordan, harmonised pathology reporting and data linkage across these pathways would materially improve the completeness of melanoma surveillance [16]. A robust diagnostic histopathology service applying contemporary WHO classification and AJCC staging, as described here, is a foundational component of that effort [6, 19].

This work also illustrates the value of laboratory-based studies in settings where population registries are still maturing. A high-volume diagnostic laboratory captures cases referred from a wide catchment of hospitals and clinics, providing a pragmatic window onto the histopathological spectrum of melanoma in the population it serves. Standardised, synoptic (structured) pathology reporting, including subtype, Breslow thickness, ulceration, and margin status, generates exactly the variables needed for staging, prognostication, and comparison with regional and international data [6, 19, 20]. We therefore recommend the routine use of synoptic melanoma reporting charts so that core data elements, in particular Breslow thickness, are captured completely for every case. As awareness and access improve, longitudinal analysis of such laboratory archives can help track shifts in incidence, subtype distribution, and depth at presentation over time [2, 4, 20].

This study has several limitations. It is a retrospective series from a single private diagnostic laboratory, so the cohort reflects that laboratory’s referral pattern, may carry referral and selection bias, and may not represent melanoma across the whole Palestinian population, particularly as some patients are diagnosed or treated abroad. Key prognostic data, most importantly Breslow thickness but also definitive subtype, ulceration, lymph node status, AJCC stage, and survival, were incompletely recorded across the archival period; depth therefore rests on the Clark level, which is not part of AJCC staging and is prognostically inferior to Breslow thickness. Because melanoma is uncommon here, the reporting pathologists’ limited routine exposure could in principle have influenced diagnostic thresholds, although the consistent immunohistochemical panel (S100, SOX10, HMB-45, and Melan-A) mitigates this. The small number of subtyped cases precludes firm conclusions about subtype prevalence, and the faster growth of nodular melanoma may have favoured its detection. These constraints, which we have reflected in our wording, reinforce the value of prospective, registry-linked studies with synoptic reporting and of systematic slide re-review to complete missing data.

Taken together, the present data position melanoma in the Palestinian population within a coherent regional pattern: low relative frequency, a slight female predominance, a substantial contribution of acral and mucosal subtypes, a predominantly nodular phenotype among the classified cases, and deep invasion at diagnosis. They provide a baseline against which future, larger, and registry-linked studies can be measured, and they highlight concrete, achievable priorities, awareness, acral and mucosal examination, immunohistochemically supported diagnosis, and complete cancer registration, for melanoma control in Palestine [12, 13, 15, 16].

## Conclusions

Cutaneous and mucosal melanoma in this Palestinian cohort were characterised by a slight female predominance, a high proportion of acral and mucosal tumours, a predominantly nodular phenotype among the cases with a recorded subtype, and deep invasion at presentation, a profile that mirrors neighbouring Arab and Eastern Mediterranean populations and differs from that of more lightly pigmented (Fitzpatrick I–II) Western series. These observations argue for strengthened melanoma awareness among the public and clinicians, routine examination of acral and mucosal sites, immunohistochemically confirmed histopathological diagnosis with contemporary subtyping and staging, and improved, linked cancer registration. Larger multicentre and registry-based studies are warranted to define incidence trends and outcomes for melanoma in the Palestinian population.

## Data Availability

The de-identified, case-level dataset analysed during the current study is available from the corresponding authors on reasonable request.

## Abbreviations

AJCC: American Joint Committee on Cancer
GLOBOCAN: Global Cancer Observatory
H&E: Haematoxylin and eosin
IHC: Immunohistochemistry
IRB: Institutional Review Board
MENA: Middle East and North Africa
SD: Standard deviation
UV: Ultraviolet
WHO: World Health Organization
